# National, Regional, State, and Selected Local Area Vaccination Coverage Among Adolescents Aged 13–17 Years — United States, 2014

**DOI:** 10.15585/mmwr.mm6429a3

**Published:** 2015-07-31

**Authors:** Sarah Reagan-Steiner, David Yankey, Jenny Jeyarajah, Laurie D. Elam-Evans, James A. Singleton, C. Robinette Curtis, Jessica MacNeil, Lauri E. Markowitz, Shannon Stokley

**Affiliations:** 1 Immunization Services Division, National Center for Immunization and Respiratory Diseases, CDC; 2 Division of Bacterial Diseases, National Center for Immunization and Respiratory Diseases, CDC; 3 Division of Sexually Transmitted Diseases, National Center for HIV/AIDS, Viral Hepatitis, STD, and TB Prevention, CDC

Routine immunization is recommended for adolescents aged 11–12 years by the Advisory Committee on Immunization Practices (ACIP) for protection against diseases including pertussis, meningococcal disease, and human papillomavirus (HPV)–associated cancers (1). To assess vaccination coverage among adolescents, CDC analyzed data collected regarding 20,827 adolescents through the 2014 National Immunization Survey–Teen (NIS-Teen).* From 2013 to 2014, coverage among adolescents aged 13–17 years increased for all routinely recommended vaccines: from 84.7% to 87.6% for ≥1 tetanus-diphtheria-acellular pertussis (Tdap) vaccine dose, from 76.6% to 79.3% for ≥1 meningococcal conjugate (MenACWY) vaccine dose, from 56.7% to 60.0% and from 33.6% to 41.7% for ≥1 HPV vaccine dose among females and males, respectively.† Coverage differed by state and local area. Despite overall progress in vaccination coverage among adolescents, HPV vaccination coverage continues to lag behind Tdap and MenACWY coverage at state and national levels. Seven public health jurisdictions achieved significant increases in ≥1- or ≥3-dose HPV vaccination coverage among females in 2014, demonstrating that substantial improvement in HPV vaccination coverage is feasible.

NIS-Teen monitors vaccination coverage among adolescents aged 13–17 years in the 50 states, District of Columbia (DC), selected local areas, and territories§ using a random-digit–dialed sample of landline and cell phone numbers.¶ NIS-Teen occurs in two phases: 1) a telephone interview with an adolescent’s parent or guardian, during which sociodemographic and vaccination provider contact information is collected and, after receiving consent, 2) a mailed questionnaire to identified vaccination providers to obtain immunization information from medical records.** Coverage estimates are based on provider-reported vaccination histories for adolescents with adequate provider data. In 2014, national estimates included information from 20,827 adolescents (10,084 females and 10,743 males).†† Details regarding NIS-Teen methodology, including methods for weighting and synthesizing provider-reported vaccination histories have been described previously (ftp://ftp.cdc.gov/pub/Health_Statistics/NCHS/Dataset_Documentation/NIS/NISPUF13_DUG.PDF ).

Revised methods for defining adequate provider data were implemented in 2014 and were retrospectively applied to 2013 NIS-Teen data for purposes of comparing these two most recent survey years. As a result, revised 2013 coverage estimates presented in this report differ from those previously published, and 2014 and revised 2013 NIS-Teen coverage estimates are not directly comparable to those published for the 2006–2013 survey years. This definition change will decrease some vaccination coverage estimates, particularly for some states and local areas. Details regarding this methodologic change and the assessment of its impact on vaccination coverage estimates are described elsewhere.† For all vaccines included in this report, t-tests were used to assess vaccination coverage differences by survey year (2014 compared with 2013), age, sex, race/ethnicity, and poverty status. Differences were considered statistically significant at p<0.05.

## National Vaccination Coverage

**FIGURE 1:**
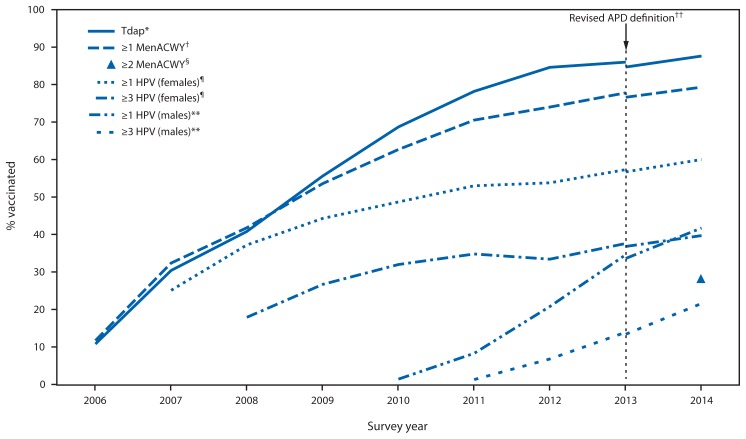
Estimated vaccination coverage with selected vaccines and doses among adolescents aged 13–17 years, by survey year — National Immunization Survey–Teen, United States, 2006–2014 Abbreviations: Tdap = tetanus toxoid, reduced diphtheria toxoid, and acellular pertussis; MenACWY = meningococcal conjugate; HPV = human papillomavirus; ACIP = Advisory Committee on Immunization Practices; APD = adequate provider data. * =1 dose Tdap vaccine at or after age 10 years. ^†^ =1 dose MenACWY or meningococcal-unknown type vaccine. ^§^ =2 doses MenACWY or meningococcal-unknown type vaccine, calculated only among adolescents aged 17 years at time of interview. Does not include adolescents who received their first and only dose of MenACWY at age 16 years or later. ^¶^HPV vaccine, either bivalent (2vHPV) or quadrivalent (4vHPV), among females. ACIP recommends 2vHPV, 4vHPV, or nine-valent (9vHPV) vaccine for females. Although the 9vHPV vaccine was licensed in December 2014 and recommended by ACIP in February 2015, it was not distributed until 2015 and thus was not administered to adolescents in 2014. ** HPV vaccine, either 2vHPV or 4vHPV, among males. ACIP recommends the 4vHPV or 9vHPV vaccines for males; however, some males might have received the 2vHPV vaccine. Although the 9vHPV vaccine was licensed in December 2014 and recommended by ACIP in February 2015, it was not distributed until 2015 and thus was not administered to adolescents in 2014. ^††^ NIS-Teen implemented a revised APD definition in 2014 and retrospectively applied the revised APD definition to 2013 data. Estimates using different APD definitions might not be directly comparable.

Compared with revised 2013 estimates, coverage among adolescents aged 13–17 years significantly increased during 2014 for Tdap, MenACWY and for each HPV dose among females and males (Table 1). Percentage point increases in coverage estimates were similar for ≥1 Tdap, ≥1 MenACWY, and, among females, ≥1 and ≥3 HPV doses (Figure 1, Table 1). Among males, coverage for ≥1 and ≥3 HPV doses increased approximately 8 percentage points from 2013 to 2014. In 2014, coverage with ≥2 MenACWY among adolescents aged 17 years was 28.5%; an additional 4.5% (95% confidence interval [CI] = 3.6%– 5.5%) of adolescents aged 17 years received their first MenACWY dose on or after their 16th birthday.

## Vaccination Coverage by Selected Characteristics

In 2014, HPV coverage and series completion were higher among older females compared with females aged 13 years; these findings were observed less consistently among males (Table 1). Vaccination coverage with each HPV dose and HPV series completion§§ were higher among females than males (Table 1). No significant differences were observed in Tdap or MenACWY vaccination coverage by sex.

Coverage estimates for each HPV dose and for ≥1 MenACWY were higher among Hispanic adolescents compared with non-Hispanic white adolescents, and estimates for each HPV dose were higher among adolescents living below the poverty level compared with those at or above the poverty level¶¶ (Table 2). Coverage with ≥1 HPV dose was higher among non-Hispanic black and American Indian/Alaska Native adolescents compared with non-Hispanic white adolescents. Similar to 2013, non-Hispanic black female adolescents had lower HPV series completion compared with non-Hispanic white female adolescents (3). Adolescents living below the poverty level had lower ≥1 Tdap coverage than adolescents living at or above the poverty level.

## State Vaccination Coverage

**FIGURE 2:**
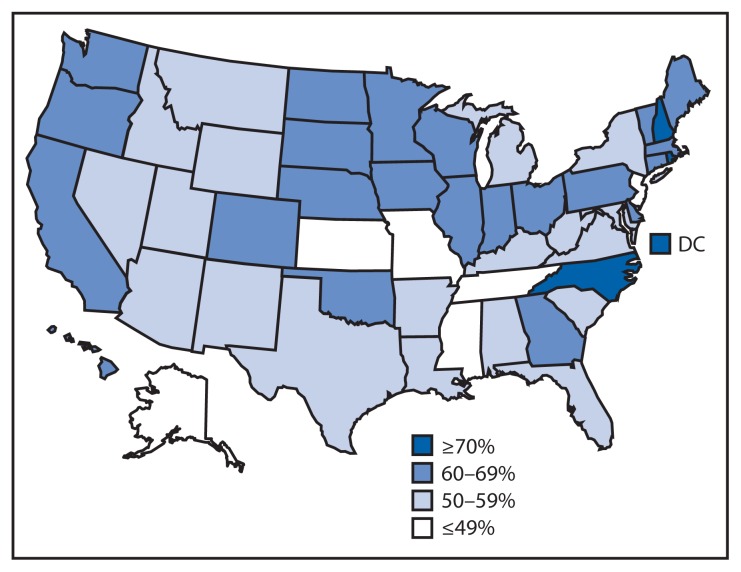
Estimated vaccination coverage with ≥1 dose of human papillomavirus (HPV) vaccine* among females aged 13–17 years^†^ — United States, National Immunization Survey–Teen, 2014 * HPV vaccine, either quadrivalent or bivalent. ^†^ Includes females (N = 10,084) born during the period January 1996–February 2002.

**FIGURE 3:**
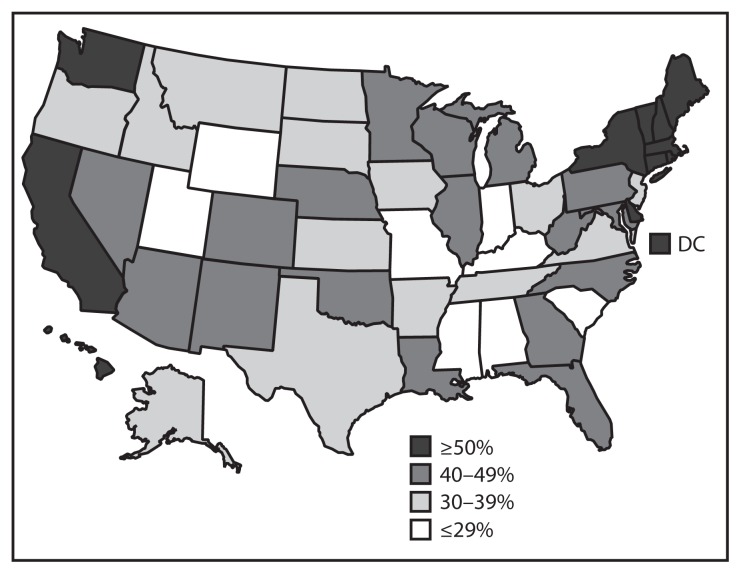
Estimated vaccination coverage with ≥1 dose of human papillomavirus (HPV) vaccine* among males aged 13–17 years^†^ — United States, National Immunization Survey–Teen, 2014 * HPV vaccine, either quadrivalent or bivalent. ^†^ Includes males (N = 10,743) born during the period January 1996–February 2002.

In 2014, vaccination coverage varied among the 50 states and DC (Table 3, Figures 2 and 3). Coverage for ≥1 Tdap dose ranged from 94.8% (Connecticut) to 70.8% (Idaho and Mississippi) and for ≥1 MenACWY dose from 95.2% (Pennsylvania) to 46.0% (Mississippi). Among females, coverage for ≥1 HPV dose ranged from 76.0% (Rhode Island) to 38.3% (Kansas) and for ≥3 HPV doses from 56.9% (DC) to 20.1% (Tennessee). In Puerto Rico, coverage with ≥1 HPV dose among females was 76.1%. Among local areas, Philadelphia, Pennsylvania, had the highest ≥1 HPV dose (80.3%) and ≥3 HPV doses (59.3%) coverage among females. Coverage with ≥1 HPV dose among females increased in six jurisdictions (Chicago, Illinois; DC; Illinois; Montana; North Carolina; and Utah) from 2013 to 2014, with percentage point increases ranging from 13.2 (Illinois) to 22.8 (DC). Coverage with ≥3 HPV doses among females increased in six jurisdictions (Chicago, Illinois; DC; Georgia; Illinois; Montana; and North Carolina); percentage point increases ranged from 14.5 (Georgia) to 28.6 (DC). One state (Tennessee) experienced a decrease (16.0 percentage points) in ≥3-dose HPV coverage among females.

### Discussion

From 2013 to 2014, vaccination coverage among adolescents aged 13–17 years increased for all vaccines routinely recommended for adolescents. Achieving high HPV vaccination coverage in early adolescence is important to optimize protection before HPV exposure. In 2014, the President’s Cancer Panel Report called for coordinated efforts to improve HPV vaccination coverage, including reducing missed opportunities to recommend and administer HPV vaccine at every clinical opportunity, increasing parents’ and adolescents’ acceptance of HPV vaccine, and maximizing access to HPV vaccination services (4).

After experiencing no progress in national HPV vaccination coverage among females aged 13–17 years from 2011 to 2012, coverage increased modestly in 2013, and an additional 3.3 percentage points in 2014 (3,5). Five states, DC, and one local area experienced large, significant increases in ≥1- or ≥3-dose HPV vaccination coverage among females, including four (Chicago, DC, Georgia, and Utah) of the 11 jurisdictions that received resources in 2013 through the Prevention and Public Health Fund from CDC to conduct activities to improve HPV vaccination coverage (6).

In six of the seven jurisdictions with increases in ≥1- or ≥3-dose HPV coverage among females, combinations of strategies were important. Immunization programs highlighted incorporating HPV vaccination in cancer control plans, joint initiatives with cancer prevention and immunization stakeholders, public communication campaigns, immunization information system–based reminder/recall, assessment and feedback activities (including clinician-to-clinician educational sessions emphasizing providing strong vaccination recommendations at ages 11–12 years), practice-focused strategies to educate staff and provide input on how to improve routine HPV vaccination within the practice, and using all opportunities to educate clinicians and parents about the importance of on-time HPV vaccination. These experiences are informing development of best practices for improving HPV vaccination coverage. At the start of 2014, only two jurisdictions had school requirements for HPV vaccination, both with broad exemption provisions (http://www.immunize.org/laws). In late 2014, DC expanded its existing school requirement for HPV vaccination to include males and females through 12th grade, with a requirement for submitting exemption forms annually (http://www.dcregs.dc.gov/Gateway/NoticeHome.aspx?NoticeID=5225019).

Some providers delay strongly recommending HPV vaccine until older adolescence (7). A comparison of age-specific HPV vaccination coverage estimates from 2013 and 2014 showed no improvement in coverage among females aged 13 years, although coverage among males aged 13 years did increase by 6.5 percentage points. Clinician resources to facilitate age-appropriate recommendation and administration of HPV vaccine are available at http://www.cdc.gov/vaccines/who/teens/for-hcp/hpv-resources.html. Changes in clinical practice, health systems, and parental acceptance take time. Because NIS-Teen monitors coverage among adolescents aged 13–17 years, the impact of interventions aimed at increasing HPV vaccine administration to adolescents aged 11–12 years cannot be measured until 1–2 years after implementation.

Estimated coverage with ≥1 MenACWY dose continues to increase among adolescents, but geographic disparities are evident and vaccination coverage estimates are still lower than for Tdap. Although 78.8% of adolescents aged 17 years received ≥1 dose of MenACWY, only 28.5% received the complete the 2-dose series. Further evaluation might identify factors that could lead to improved MenACWY series coverage, although older adolescents have fewer preventive health visits, and awareness of the 2-dose recommendation (http://www.cdc.gov/mmwr/preview/mmwrhtml/mm6003a3.htm) might still be low. In addition, because NIS-Teen includes adolescents aged 13–17 years, receipt of MenACWY at age ≥18 years is not captured in these coverage estimates.

MMR vaccine is routinely recommended at ages 12–15 months and 4–6 years (1), and although ≥2-dose MMR coverage among adolescents remains high nationally, seven states had coverage <90%,*** suggesting important vulnerability to measles outbreaks. As of July 24, 2015, a total of 183 measles cases have been reported this year in the United States (http://www.cdc.gov/measles/cases-outbreaks.html). High MMR coverage is needed to sustain elimination and protect those who cannot be directly vaccinated. Health care providers of adolescents should assess their patients’ vaccination status at each clinical opportunity, take advantage of immunization information systems, which should reflect vaccines delivered in any setting, and offer all vaccines for which adolescents are eligible, including missing doses of MMR, varicella, and hepatitis B vaccines.

The findings in this report are subject to at least three limitations. First, household response rates for landline and cell phone samples were 60.3% and 31.2%, respectively, and only 57.1% of landline-completed interviews and 52.3% of cell phone–completed interviews had adequate provider data. Second, estimates might be biased even after adjustments for nonresponse and phoneless households. A total survey error model of 2011 NIS-Teen that included comparison with provider-reported data from National Health Interview Survey participants indicated coverage estimates were 1.3–6.7 percentage points higher as a result of noncoverage and household nonresponse error.††† Weights have been adjusted to account for the increasing prevalence of cell phone–only households over time. Nonresponse bias might change, which could affect comparisons of estimates across survey years. Finally, estimates stratified by state/local area and race/ethnicity might be unreliable because of small sample sizes.

National HPV vaccination coverage estimates continue to be low for adolescents, despite similar percentage point increases in coverage in 2014 for ≥1 Tdap dose, ≥1 MenACWY dose, and, among females, ≥1 HPV dose. Differences in coverage estimates by vaccine indicate many missed opportunities for simultaneous administration of HPV with Tdap or MenACWY. Wide state and local variation in adolescent coverage with routinely recommended vaccines persists. Routinely recommending HPV vaccination at ages 11–12 years during the same visit and with the same emphasis used for other vaccines is critical. Resources are available for clinicians that focus on cancer prevention and ways to confidently address questions regarding HPV vaccine safety and efficacy. Multifaceted interventions that engage clinicians and other immunization stakeholders and increase community awareness might improve HPV vaccination coverage (8). Recent licensure of two vaccines for adolescents (nine-valent HPV [9vHPV] and serogroup B meningococcal vaccines) might provide opportunities for additional protection of adolescents against vaccine-preventable diseases in the years ahead (2,9). Furthermore, clinical trials are ongoing to evaluate alternative dosing schedules for 9vHPV, which will be reviewed by ACIP in consideration of reduced-dose HPV vaccination schedules in the United States (2). To protect against HPV-associated cancers and other vaccine-preventable diseases, clinicians should ensure that adolescents receive all vaccines currently recommended routinely at ages 11–12 years.

**Summary**

What is already known on this topic?

Routine immunization is recommended for adolescents aged 11–12 years by the Advisory Committee on Immunization Practices for protection against diseases including pertussis, meningococcal disease, and human papillomavirus (HPV)–associated cancers. During 2006–2013, national coverage with ≥1 dose of tetanus-diphtheria-acellular pertussis (Tdap) vaccine and ≥1 dose of meningococcal conjugate (MenACWY) vaccine increased annually. Although ≥1-dose HPV coverage among females increased during 2007–2011, no change was observed during 2011–2012. However, during 2012–2013 and 2011–2013, ≥1-dose HPV coverage among females and males, respectively, increased.

What is added by this report?

During 2013–2014, vaccination coverage among adolescents aged 13–17 years increased for ≥1 dose of Tdap, ≥1 dose of MenACWY, and each HPV dose among females and males, with considerable variation in coverage by state. Although HPV vaccination coverage among females increased nationally for the second consecutive year, HPV coverage lags behind Tdap and MenACWY coverage. Seven jurisdictions achieved significant increases in ≥1- or ≥3-dose HPV vaccination coverage among females during 2013–2014, demonstrating that substantial improvement in HPV vaccination coverage is feasible.

What are the implications for public health practice?

Despite similar percentage point increases in coverage with Tdap and MenACWY vaccines, and ≥1 HPV dose among females in 2014, national HPV coverage estimates remain low for adolescents. Differences in coverage estimates by vaccine indicate missed opportunities for administering HPV vaccine at visits when Tdap or MenACWY vaccines are given. Routinely recommending HPV vaccine at ages 11–12 years, during the same visit and with the same emphasis used for other vaccines, is critical. Multifaceted interventions that engage clinicians and other immunization stakeholders and increase community awareness might improve HPV vaccination coverage.

## Figures and Tables

**TABLE 1:** Estimated vaccination coverage with selected vaccines and doses among adolescents aged 13–17* years, by age at interview — National Immunization Survey–Teen (NIS-Teen), United States, 2014

Vaccine	Age at interview (yrs) (2014)					Total (adolescents aged 13–17 yrs)	
	
	13 (n = 4,292) % (95% CI)	14 (n = 4,329) % (95% CI)	15 (n = 4,143) % (95% CI)	16 (n = 4,215) % (95% CI)	17 (n = 3,848) % (95% CI)	2014 (n = 20,827) % (95% CI)	2013† (n = 18,948) % (95% CI)
**Tdap**§ **≥1 dose**	87.5 (±2.1)	89.1 (±1.6)	88.3 (±1.9)	86.9 (±2.1)	86.3 (±2.0)	87.6 (±0.9)¶	84.7 (±1.0)
MenACWY** **≥1 dose**	78.0 (±2.5)	81.0 (±2.1)	79.2 (±2.5)	79.4 (±2.5)	78.8 (±2.5)	79.3 (±1.1)¶	76.6 (±1.1)
**MenACWY ≥2 doses**	—	—	—	—	28.5 (±2.8)††	—	—
**HPV**§§ **vaccine coverage by doses**							
Females							
≥1 dose	51.1 (±4.1)	56.6 (±3.9)	61.0 (±4.3)¶¶	64.4 (±4.1)¶¶	66.5 (±4.4)¶¶	60.0 (±1.9)¶	56.7 (±1.9)
≥2 doses	40.1 (±4.0)	46.4 (±4.0)¶¶	51.6 (±4.3)¶¶	55.7 (±4.2)¶¶	57.6 (±4.7)¶¶	50.3 (±1.9)¶	46.9 (±1.9)
≥3 doses	26.2 (±3.6)	35.9 (±3.9)¶¶	41.2 (±4.2)¶¶	43.8 (±4.1)¶¶	51.0 (±4.7)¶¶	39.7 (±1.9)¶	36.8 (±1.9)
Males							
≥1 dose	38.9 (±4.2)	42.6 (±4.0)	45.7 (±4.1)¶¶	40.0 (±4.0)	41.8 (±4.1)	41.7 (±1.8)¶	33.6 (±1.8)
≥2 doses	27.1 (±3.9)	30.9 (±3.8)	35.8 (±4.1)¶¶	31.2 (±3.8)	32.6 (±4.0)	31.4 (±1.7)¶	22.6 (±1.6)
≥3 doses	16.2 (±3.3)	20.9 (±3.5)	24.9 (±4.0)¶¶	22.9 (±3.5)¶¶	23.3 (±3.7)¶¶	21.6 (±1.6)¶	13.4 (±1.3)
**HPV vaccine 3-dose series completion** ***							
Females	56.1 (±6.3)	66.8 (±5.2)¶¶	70.3 (±5.0)¶¶	70.8 (±5.2)¶¶	78.3 (±5.4)¶¶	69.3 (±2.4)	69.8 (±2.5)
Males	47.1 (±7.6)	56.6 (±6.6)	58.1 (±6.6)¶¶	64.7 (±6.1)¶¶	61.7 (±6.6)¶¶	57.8 (±3.0)¶	48.2 (±3.9)
MMR ≥2 doses	90.2 (±1.8)	91.1 (±1.6)	91.2 (±1.6)	90.2 (±1.9)	90.9 (±1.6)	90.7 (±0.8)	89.6 (±0.9)
HepB ≥3 doses	91.3 (±1.8)	91.7 (±1.5)	92.5 (±1.4)	90.2 (±2.0)	91.4 (±1.5)	91.4 (±0.7)	91.3 (±0.8)
**Varicella vaccine**							
History of varicella†††	13.7 (±2.0)	17.8 (±2.4)¶¶	20.2 (±2.4)¶¶	24.2 (±2.6)¶¶	29.3 (±2.8)¶¶	21.0 (±1.1)¶	25.2 (±1.1)
**Among adolescents with no history of varicella**							
≥1 dose vaccine	95.6 (±1.3)	95.7 (±1.2)	95.6 (±1.1)	95.1 (±1.2)	93.6 (±1.5)	95.2 (±0.6)¶	93.5 (±0.9)
≥2 doses vaccine	83.1 (±2.4)	81.9 (±2.3)	81.1 (±2.6)	81.0 (±2.6)	77.1 (±3.1)¶¶	81.0 (±1.2)¶	76.8 (±1.3)
History of varicella or received ≥2 doses varicella vaccine	85.4 (±2.1)	85.1 (±1.9)	85.0 (±2.1)	85.6 (±2.0)	83.8 (±2.3)	85.0 (±0.9)¶	82.7 (±1.0)

**Abbreviations:** CI = confidence interval; Tdap = tetanus-diphtheria-acellular pertussis vaccine; MenACWY = meningococcal conjugate vaccine; HPV = human papillomavirus; MMR = measles, mumps, and rubella vaccine; HepB = hepatitis B vaccine.

* Adolescents (N = 20,827) in the 2014 NIS-Teen were born during the period January 1996-February 2002.

† Revised estimates for overall NIS-Teen data for 2013 were provided as a comparison to overall 2014 NIS-Teen data. A revised adequate provider data definition was implemented in 2014 NIS-Teen, and estimates might not be directly comparable to those previously published. For comparative purposes, 2013 estimates included in this table have been calculated by retrospectively applying the revised adequate provider data definition to 2013 NIS-Teen data and, as a result, will differ from those previously published.

§ Includes percentages receiving Tdap at or after age 10 years.

¶ Statistically significant difference (p<0.05) compared with 2013 NIS-Teen estimates.

** Includes percentages receiving MenACWY or meningococcal-unknown type vaccine.

†† ≥2 doses of MenACWY or meningococcal-unknown type vaccine. Calculated only among adolescents who were aged 17 years at time of interview. Does not include adolescents who received 1 dose of MenACWY vaccine at or after age 16 years.

§§ HPV vaccine, either quadrivalent (4vHPV) or bivalent (2vHPV). Although only 4vHPV was recommended for use in males in 2014, some might have received 2vHPV. In 2014 data, percentage was reported among 10,084 females and 10,743 males. In 2013 data, percentage was reported among 9,042 females and 9,906 males. Some adolescents might have received more than the 3 recommended HPV vaccine doses.

¶¶ Statistically significant difference (p<0.05) in estimated vaccination coverage by age; reference group was adolescents aged 13 years.

*** The completion rate for the 3-dose HPV vaccination series represents the percentage of adolescents who received ≥3 HPV doses among those who had ≥1 HPV vaccine dose with at least 24 weeks between the first dose and the interview date. The denominator for this calculation was limited to 5,703 females and 3,935 males in 2014 and 4,704 females and 2,623 males in 2013 who received their first HPV dose and had enough time to receive the third HPV dose.

††† By parent/guardian report or provider records.

**TABLE 2:** Estimated vaccination coverage among adolescents aged 13–17 years,* by race/ethnicity,† poverty level,§ and selected vaccines and doses — National Immunization Survey–Teen (NIS-Teen), United States, 2014

Vaccine	Race/Ethnicity						Poverty status	
	
	White only, non-Hispanic (n = 13,443) % (95% CI)¶	Black only, non-Hispanic (n = 1,986) % (95% CI)	Hispanic (n = 3,255) % (95% CI)	American Indian/Alaska Native only, non-Hispanic (n = 303) % (95% CI)	Asian, non-Hispanic (n = 764) % (95% CI)	Multiracial (n = 985) % (95% CI)	Below poverty level (n = 3,709) % (95% CI)	At or above poverty level (n = 16,404) % (95% CI)
**Tdap** ** **≥1 dose**	88.6 (±0.9)	87.6 (±2.1)	86.7 (±2.4)	86.1 (±6.5)	85.2 (±6.7)	81.9 (±6.3)††	85.8 (±2.0)††	88.4 (±0.9)
**MenACWY** §§ **≥1 dose**	78.2 (±1.2)	80.3 (±2.8)	82.1 (±2.8)††	73.5 (±9.2)	82.5 (±6.5)	74.3 (±6.5)	79.0 (±2.4)	79.5 (±1.2)
**HPV**¶¶ **vaccine coverage by doses**								
Females								
≥1 dose	56.1 (±2.2)	66.4 (±4.8)††	66.3 (±5.1)††	71.2 (±14.4)††	54.9 (±9.3)	55.9 (±7.5)	67.2 (±4.2)††	57.7 (±2.1)
≥2 doses	47.1 (±2.2)	53.0 (±5.1)††	57.4 (±5.1)††	61.8 (±15.6)	47.5 (±9.1)	45.5 (±7.3)	58.0 (±4.3)††	47.9 (±2.2)
≥3 doses	37.5 (±2.1)	39.0 (±5.0)	46.9 (±5.2)††	39.4 (±15.4)	35.7 (±8.2)	37.2 (±7.0)	44.7 (±4.3)††	37.9 (±2.1)
Males								
≥1 dose	36.4 (±2.0)	42.1 (±4.9)††	54.2 (±4.9)††	49.8 (±13.9)	45.8 (±11.4)	40.2 (±10.1)	51.6 (±4.0)††	39.5 (±2.1)
≥2 doses	27.4 (±1.9)	32.0 (±4.8)	39.4 (±4.9)††	40.5 (±13.1)	38.3 (±11.1)	32.4 (±9.9)	39.4 (±4.1)††	29.5 (±2.0)
≥3 doses	18.8 (±1.7)	20.4 (±4.0)	27.8 (±4.7)††	26.3 (±10.9)	26.6 (±10.4)	23.5 (±9.6)	27.2 (±3.9)††	20.2 (±1.8)
**HPV vaccine 3-dose series completion** ***								
Females	70.6 (±3.2)	61.6 (±6.3)††	72.8 (±5.4)	55.4 (±22.5)	71.7 (±11.0)	68.9 (±9.5)	68.3 (±5.0)	69.4 (±2.9)
Males	57.9 (±3.6)	54.1 (±8.1)	57.2 (±7.0)	57.7 (±17.5)	63.0 (±17.0)	65.1 (±13.6)	58.2 (±6.2)	57.4 (±3.5)
**≥2 MMR**	91.0 (±0.9)	91.1 (±1.9)	90.5 (±1.9)	94.1 (±4.1)	85.8 (±6.9)	90.0 (±3.3)	90.5 (±1.6)	90.8 (±0.9)
**≥3 HepB**	92.2 (±0.8)	91.4 (±1.8)	90.5 (±1.9)	93.9 (±4.3)	85.5 (±6.9)	90.4 (±3.4)	90.3 (±1.7)	91.9 (±0.8)
**Varicella vaccine**								
History of varicella†††	20.2 (±1.2)	18.3 (±2.8)	23.3 (±3.1)	36.1 (±11.8)††	23.2 (±7.3)	20.5 (±4.3)	24.8 (±2.6)††	19.5 (±1.2)
**Among adolescents with no history of varicella**								
≥1 dose vaccine	95.1 (±0.7)	95.3 (±1.4)	95.5 (±1.5)	96.1 (±3.4)	92.4 (±4.2)	95.5 (±2.5)	95.0 (±1.3)	95.2 (±0.6)
≥2 doses vaccine	80.0 (±1.4)	84.6 (±2.5)††	82.5 (±3.1)	84.7 (±6.7)	82.3 (±5.5)	73.1 (±7.8)	82.7 (±2.3)	80.8 (±1.3)
History of varicella or received ≥2 doses varicella vaccine	84.0 (±1.1)	87.4 (±2.1)††	86.6 (±2.4)	90.2 (±4.5)††	86.4 (±4.4)	78.6 (±6.5)	87.0 (±1.8)††	84.5 (±1.1)

**Abbreviations:** CI = confidence interval; Tdap = tetanus-diphtheria-acellular pertussis vaccine; MenACWY = meningococcal conjugate vaccine; HPV = human papillomavirus; MMR = measles, mumps, and rubella vaccine; HepB = hepatitis B vaccine.

* Adolescents (N = 20,827) in the 2014 NIS-Teen were born during the period January 1996-February 2002.

† Adolescent’s race/ethnicity was reported by their parent or guardian. Adolescents identified in this report as white, black, Asian, American Indian/Alaska Native, or multiracial were reported by the parent or guardian as non-Hispanic. Adolescents identified as multiracial had more than one race category selected. Adolescents identified as Hispanic might be of any race. Native Hawaiian or other Pacific Islanders were not included in the table because of small sample sizes.

§ Adolescents were classified as below poverty level if their total family income was less than the federal poverty level specified for the applicable family size and number of children aged <18 years. All others were classified as at or above the poverty level. Additional information available at http://www.census.gov/hhes/www/poverty/data/threshld/index.html. Poverty status was unknown for 714 adolescents.

¶ Estimates with 95% CI half-widths >10 might not be reliable.

** Includes percentages receiving Tdap at or after age 10 years.

†† Statistically significant difference (p<0.05) in estimated vaccination coverage by race/ethnicity or poverty level; referent groups were white, non-Hispanic adolescents, and adolescents living at or above poverty level, respectively.

§§ Includes percentages receiving MenACWY and meningococcal-unknown type vaccine.

¶¶ HPV vaccine, either quadrivalent (4vHPV) or bivalent (2vHPV). Although only 4vHPV was recommended for use in males in 2014, some males might have received 2vHPV. Percentage was reported among 10,084 females and 10,743 males. Some adolescents might have received more than the 3 recommended HPV vaccine doses.

*** The completion rate for the 3-dose HPV vaccination series represents the percentage of adolescents who received 3 HPV doses among those who had ≥1 HPV vaccine dose with at least 24 weeks between the first dose and the interview date. The denominator for this calculation was limited to 5,703 females and 3,935 males who received their first HPV dose and had enough time to receive the third HPV dose.

††† By parent/guardian report or provider records.

**TABLE 3:** Estimated vaccination coverage with selected vaccines and doses* among adolescents aged 13–17 years,† by HHS region and state or selected local areas — National Immunization Survey–Teen (NIS-Teen), United States, 2014

HHS region and state/local area	≥1 Tdap§ % (95% CI)¶¶	≥1 MenACWY¶ % (95% CI)	Females (N = 10,084)			Males (N = 10,743)		
	
			≥1 HPV** % (95% CI)	≥2 HPV†† % (95% CI)	≥3 HPV§§ % (95% CI)	≥1 HPV** % (95% CI)	≥2 HPV†† % (95% CI)	≥3 HPV§§ % (95% CI)
**United States overall**	**87.6 (±0.9)** ***	**79.3 (±1.1)** ***	**60.0 (±1.9)** ***	**50.3 (±1.9)** ***	**39.7 (±1.9)** ***	**41.7 (±1.8)** ***	**31.4 (±1.7)** ***	**21.6 (±1.6)** ***
**HHS Region I**	93.0 (±1.8)	90.8 (±1.8)***	67.8 (±4.6)	61.0 (±4.8)***	49.0 (±5.0)***	54.1 (±4.7)	44.4 (±4.7)***	29.0 (±4.2)***
Connecticut	94.8 (±3.2)	94.9 (±3.0)	63.5 (±8.5)	59.9 (±8.7)	48.5 (±9.1)	50.3 (±9.0)	38.4 (±8.7)	27.0 (±7.8)
Maine	85.4 (±4.7)	73.6 (±5.7)	66.8 (±8.1)	52.9 (±8.7)	43.0 (±8.6)	53.1 (±9.0)	42.5 (±8.8)	27.5 (±7.6)***
Massachusetts	93.2 (±3.4)	92.1 (±3.3)	69.0 (±8.5)	62.5 (±9.0)***	49.5 (±9.2)	54.3 (±8.5)	46.2 (±8.6)	27.3 (±7.7)
New Hampshire	94.4 (±2.6)	90.6 (±3.2)	71.0 (±7.2)	61.2 (±7.9)	50.1 (±8.4)	56.1 (±7.8)***	46.9 (±7.9)***	33.0 (±7.6)***
Rhode Island	92.4 (±3.4)	94.1 (±3.2)	76.0 (±7.7)	67.8 (±8.2)	53.7 (±8.5)	69.0 (±7.5)	56.8 (±8.1)	42.9 (±7.9)
Vermont	93.4 (±3.3)	81.3 (±5.1)	63.4 (±8.9)	55.8 (±9.2)	49.8 (±9.2)	50.5 (±9.3)	40.5 (±9.1)***	30.5 (±8.4)
**HHS Region II**	91.0 (±2.4)	84.6 (±3.0)	55.3 (±5.9)	44.8 (±5.9)	38.3 (±5.8)	45.1 (±5.6)***	34.5 (±5.3)***	26.1 (±5.1)***
New Jersey	90.1 (±4.4)	94.9 (±3.2)	48.0 (±9.8)	39.9 (±9.6)	34.5 (±9.3)	35.5 (±9.4)	26.7 (±8.7)	21.2 (±8.4)
New York	91.5 (±2.8)	79.6 (±4.2)	58.8 (±7.4)	47.2 (±7.5)	40.1 (±7.3)	49.8 (±6.8)***	38.2 (±6.7)***	28.5 (±6.3)***
NY-City of New York	88.7 (±4.9)	86.8 (±4.9)	58.0 (±10.2)	46.2 (±10.2)	38.3 (±9.9)	56.6 (±9.8)	46.3 (±10.2)	35.0 (±10.0)
NY-Rest of state	93.2 (±3.5)	75.1 (±6.0)	59.3 (±10.2)	47.8 (±10.3)	41.2 (±10.1)	45.5 (±9.1)***	33.1 (±8.7)	24.4 (±8.1)***
**HHS Region III**	89.8 (±1.9)***	85.9 (±2.3)***	62.5 (±4.8)***	54.3 (±4.9)	42.5 (±4.8)	44.4 (±4.9)***	34.5 (±4.6)***	24.8 (±4.2)***
Delaware	90.5 (±3.7)***	86.7 (±4.6)	67.6 (±9.3)	51.4 (±9.9)	42.3 (±9.8)	54.6 (±9.5)***	43.8 (±9.9)***	31.0 (±9.7)***
District of Columbia	81.4 (±5.9)	93.5 (±2.8)	75.2 (±9.4)***	67.8 (±10.3)***	56.9 (±10.9)***	68.1 (±9.5)	54.3 (±10.9)	34.5 (±11.0)
Maryland	85.0 (±5.3)	86.5 (±4.9)***	57.9 (±9.9)	52.6 (±10.0)	39.4 (±9.7)	46.9 (±9.7)***	37.3 (±9.4)***	24.5 (±8.6)
Pennsylvania	93.0 (±2.7)	95.2 (±1.9)***	66.8 (±7.4)	57.9 (±8.0)	48.2 (±8.1)	47.4 (±7.9)	35.9 (±7.4)	26.0 (±6.7)***
PA-Philadelphia	90.3 (±4.3)	92.6 (±3.7)	80.3 (±8.1)	74.1 (±8.8)	59.3 (±10.1)	62.8 (±9.1)	49.9 (±9.5)***	34.8 (±8.9)***
PA-Rest of state	93.4 (±2.9)	95.6 (±2.1)***	65.1 (±8.4)	55.7 (±9.0)	46.7 (±9.1)	45.4 (±8.9)	34.1 (±8.3)	24.9 (±7.4)
Virginia	91.2 (±3.9)***	72.5 (±6.6)	59.2 (±10.4)	51.1 (±10.5)	35.9 (±9.7)	36.3 (±10.5)	29.7 (±9.9)	22.5 (±9.4)
West Virginia	77.9 (±5.8)	78.9 (±5.6)	58.0 (±9.4)	48.3 (±9.3)	40.0 (±9.0)	42.7 (±8.9)***	28.8 (±8.0)	23.5 (±7.7)
**HHS Region IV**	86.8 (±1.8)***	71.8 (±2.6)	58.4 (±4.0)***	46.3 (±4.1)	36.5 (±3.9)	36.7 (±3.9)***	25.6 (±3.6)***	16.7 (±3.1)***
Alabama	88.6 (±4.0)	71.6 (±5.7)	54.7 (±9.3)	40.7 (±9.0)	35.3 (±8.8)	27.6 (±7.2)	16.1 (±5.8)	9.0 (±4.7)
Florida	90.7 (±4.2)	72.2 (±6.7)	57.2 (±10.4)	39.6 (±10.0)	28.5 (±9.1)	41.0 (±10.1)	30.0 (±9.5)***	17.5 (±8.1)
Georgia	86.1 (±4.8)	74.9 (±6.1)	65.4 (±9.1)	56.3 (±9.5)***	47.1 (±9.7)***	41.2 (±9.0)	28.0 (±7.8)	21.0 (±7.2)
Kentucky	85.5 (±4.8)	78.2 (±5.7)	52.1 (±9.5)	45.1 (±9.4)	37.5 (±9.2)	23.7 (±8.0)	17.5 (±7.2)	13.3 (±6.6)
Mississippi	70.8 (±6.3)***	46.0 (±6.5)	45.8 (±9.5)	30.6 (±8.7)	24.6 (±8.4)	26.5 (±8.0)***	16.2 (±7.0)	NA
North Carolina	92.3 (±3.7)	74.1 (±5.6)	71.1 (±8.1)***	60.0 (±9.0)***	54.0 (±9.2)***	45.2 (±8.9)	31.9 (±8.4)	20.9 (±7.3)
South Carolina	72.6 (±6.2)	67.3 (±6.3)	52.1 (±9.5)	46.5 (±9.5)	35.9 (±9.1)	29.4 (±8.5)	22.5 (±7.8)***	16.1 (±6.8)
Tennessee	86.0 (±4.5)***	74.0 (±5.8)	47.8 (±9.8)	39.4 (±9.6)	20.1 (±6.7)***	30.5 (±8.5)	19.4 (±7.2)	14.0 (±6.6)
**HHS Region V**	86.7 (±1.8)	80.1 (±2.1)	61.9 (±3.5)***	52.7 (±3.7)***	41.9 (±3.6)***	39.6 (±3.5)***	31.2 (±3.4)***	20.6 (±3.0)***
Illinois	91.9 (±2.4)***	77.1 (±4.2)	64.4 (±6.5)***	58.0 (±6.7)***	47.7 (±6.9)***	44.7 (±6.6)***	34.2 (±6.3)***	22.6 (±5.7)
IL-City of Chicago	84.6 (±5.8)	83.4 (±5.9)	78.1 (±8.1)***	68.8 (±9.5)***	52.6 (±10.7)***	64.9 (±10.0)***	44.3 (±10.8)***	26.1 (±9.3)
IL-Rest of state	93.6 (±2.6)***	75.6 (±5.0)	61.2 (±7.7)	55.5 (±8.0)***	46.5 (±8.2)***	40.0 (±7.6)	31.9 (±7.3)***	21.8 (±6.6)
Indiana	88.6 (±4.1)	90.0 (±3.9)	61.4 (±8.5)	54.3 (±8.9)	44.4 (±9.0)	23.2 (±6.9)	17.0 (±5.9)	12.8 (±5.1)
Michigan	79.3 (±5.4)	90.7 (±4.0)	58.0 (±9.1)	50.9 (±9.3)	40.9 (±9.1)	39.8 (±9.5)	31.9 (±9.1)***	22.1 (±8.2)
Minnesota	87.2 (±5.0)	75.5 (±6.0)	67.0 (±9.4)	53.9 (±10.3)	42.5 (±10.3)	43.9 (±9.9)***	36.6 (±9.8)***	13.6 (±7.0)
Ohio	83.0 (±4.8)	73.7 (±5.4)	61.0 (±8.4)	47.3 (±8.8)	35.2 (±8.3)	36.8 (±8.1)	29.3 (±7.7)	23.3 (±7.3)
Wisconsin	93.3 (±3.7)	73.8 (±6.2)	61.0 (±9.8)	52.1 (±10.0)	40.9 (±9.9)	49.3 (±9.4)***	39.3 (±9.5)***	23.6 (±8.1)***
**HHS Region VI**	87.8 (±2.2)***	85.0 (±2.2)***	53.0 (±5.0)	44.6 (±5.0)	34.2 (±4.7)	38.2 (±4.6)	27.1 (±4.2)	18.2 (±3.8)
Arkansas	84.6 (±4.7)***	64.8 (±6.1)***	54.6 (±9.1)	37.8 (±8.7)	23.4 (±7.5)	35.1 (±8.9)***	21.8 (±7.8)***	11.4 (±5.6)
Louisiana	93.8 (±2.8)***	91.8 (±3.4)	53.2 (±9.5)	43.8 (±9.2)	38.4 (±9.0)	44.7 (±9.2)***	32.2 (±8.6)	21.5 (±7.6)
New Mexico	83.3 (±5.3)	75.1 (±5.6)	59.0 (±8.9)	48.7 (±9.1)	39.9 (±8.9)	42.8 (±9.3)	33.2 (±8.9)	23.3 (±8.1)
Oklahoma	82.6 (±4.7)	70.8 (±5.8)	65.3 (±8.6)	50.8 (±9.3)	36.4 (±9.1)	43.2 (±8.9)	30.2 (±8.2)	19.9 (±7.1)
Texas	88.2 (±3.1)	88.6 (±3.0)	50.7 (±7.0)	44.2 (±6.9)	33.9 (±6.5)	36.6 (±6.4)	26.0 (±5.8)	17.7 (±5.2)
TX-Bexar County	85.7 (±4.3)	84.3 (±5.0)	47.7 (±9.4)	39.0 (±9.1)	30.8 (±8.5)	35.6 (±8.8)	26.2 (±8.5)	15.0 (±6.7)
TX-City of Houston	87.8 (±4.6)	87.4 (±5.0)	66.8 (±9.0)	55.2 (±9.6)	43.8 (±9.8)	53.7 (±9.9)***	38.6 (±9.6)***	27.1 (±9.0)
TX-El Paso County	86.3 (±5.1)	91.7 (±4.1)	71.9 (±9.9)	61.7 (±10.7)	45.6 (±11.0)	54.2 (±10.3)	42.9 (±10.2)	31.8 (±9.7)
TX-Rest of state	88.5 (±3.6)	88.9 (±3.5)	48.7 (±8.2)	43.0 (±8.2)	32.8 (±7.7)	34.4 (±7.5)	24.2 (±6.8)	16.5 (±6.1)
**HHS Region VII**	82.1 (±2.8)	65.4 (±3.6)	49.8 (±5.2)	40.6 (±5.0)	31.6 (±4.6)	31.0 (±4.7)	23.8 (±4.3)***	16.3 (±3.5)***
Iowa	76.7 (±6.4)	64.4 (±6.9)	59.5 (±9.9)	52.5 (±9.9)	37.6 (±9.3)	30.2 (±8.8)	26.7 (±8.5)	18.7 (±7.3)
Kansas	79.8 (±5.6)	65.1 (±6.5)***	38.3 (±9.5)	30.4 (±8.7)	24.8 (±8.0)	32.8 (±8.6)	23.5 (±7.7)	19.5 (±7.4)
Missouri	86.1 (±4.6)	63.3 (±6.5)	47.5 (±9.2)	36.3 (±8.8)	28.3 (±8.2)	27.9 (±8.4)	20.1 (±7.6)***	11.3 (±5.7)
Nebraska	82.2 (±5.4)	74.1 (±5.8)	59.6 (±9.1)	51.2 (±9.4)	43.3 (±9.5)	39.5 (±9.1)	31.0 (±8.8)	22.8 (±7.8)
**HHS Region VIII**	87.1 (±2.2)	70.9 (±3.0)***	60.3 (±4.6)***	48.5 (±4.8)	36.2 (±4.6)	35.2 (±4.5)***	25.7 (±4.2)***	18.1 (±3.7)***
Colorado	90.2 (±3.6)	76.8 (±4.9)	62.5 (±8.3)	55.1 (±8.7)	42.1 (±8.7)	40.7 (±8.2)	30.8 (±7.8)	21.9 (±7.0)***
Montana	84.7 (±4.7)	60.2 (±6.5)***	57.2 (±9.2)***	51.0 (±9.2)***	42.9 (±9.1)***	33.3 (±9.2)	19.1 (±7.6)	13.0 (±6.4)
North Dakota	92.1 (±4.0)	91.8 (±3.3)	60.9 (±9.4)	48.7 (±9.6)	41.7 (±9.4)	37.6 (±9.0)	32.1 (±8.4)	25.3 (±7.8)
South Dakota	75.0 (±5.9)	57.0 (±6.6)	61.0 (±9.4)	44.0 (±9.5)	33.1 (±8.8)	34.4 (±9.1)***	28.4 (±8.8)***	23.5 (±8.5)***
Utah	84.8 (±4.5)	66.9 (±5.9)	59.2 (±8.3)***	40.0 (±8.5)	26.0 (±7.3)	28.6 (±8.0)***	19.6 (±6.8)***	12.4 (±5.5)
Wyoming	89.1 (±3.5)	55.6 (±5.7)	50.3 (±8.1)	42.4 (±8.0)	33.6 (±7.6)	29.3 (±7.4)***	19.3 (±6.6)	12.2 (±5.5)
**HHS Region IX**	87.1 (±3.7)	79.5 (±4.5)	66.7 (±7.5)	58.0 (±7.8)	45.0 (±7.8)	50.2 (±7.4)	38.8 (±7.3)	28.2 (±7.1)***
Arizona	84.2 (±4.8)	85.9 (±4.7)	58.2 (±9.4)	46.2 (±9.4)	35.8 (±8.8)	40.6 (±8.3)	28.2 (±7.5)	16.7 (±5.7)
California	87.7 (±4.6)	79.3 (±5.7)	69.2 (±9.4)	61.5 (±9.8)	47.7 (±9.8)	52.1 (±9.3)	41.2 (±9.2)	31.1 (±8.9)***
Hawaii	82.3 (±4.8)	77.7 (±5.2)	60.4 (±8.6)	49.3 (±8.7)	38.0 (±8.4)	56.5 (±8.6)***	47.1 (±8.8)***	30.9 (±8.5)***
Nevada	87.6 (±3.8)	66.5 (±5.9)	54.2 (±8.6)	43.5 (±8.5)	32.5 (±8.2)	43.4 (±9.0)	28.3 (±8.2)	15.7 (±6.0)***
**HHS Region X**	85.1 (±2.6)	76.2 (±3.2)***	63.6 (±5.4)	52.9 (±5.7)	42.3 (±5.7)	45.0 (±5.4)***	32.9 (±5.2)***	19.5 (±4.5)***
Alaska	73.8 (±5.4)	56.9 (±6.1)	48.7 (±8.8)	45.2 (±8.7)	34.4 (±8.2)	37.9 (±8.6)	25.9 (±7.8)	13.3 (±6.3)
Idaho	70.8 (±6.4)	78.1 (±5.8)	59.4 (±10.2)	54.2 (±10.2)	38.3 (±9.9)	32.0 (±8.7)	22.8 (±7.9)	17.2 (±6.9)
Oregon	88.0 (±4.2)	68.4 (±6.0)	64.6 (±8.7)	51.7 (±9.2)	43.1 (±9.1)	36.9 (±8.4)	23.0 (±7.0)	12.3 (±4.8)
Washington	88.5 (±4.1)	82.1 (±4.9)	65.8 (±8.8)	54.1 (±9.3)	43.8 (±9.4)	53.8 (±8.8)***	41.8 (±8.8)***	24.6 (±7.9)***
*Range* †††	*(70.8–94.8)*	*(46.0–95.2)*	*(38.3–76.0)*	*(30.4–67.8)*	*(20.1–56.9)*	*(23.2–69.0)*	*(16.1–56.8)*	*(9.0–42.9)*
**Territory**								
Puerto Rico	81.7 (±7.2)	83.5 (±6.7)	76.1 (±10.4)	60.7 (±12.8)	49.9 (±13.0)	54.3 (±12.5)	41.6 (±12.5)	23.7 (±10.9)

**Abbreviations:** CI = confidence interval; Tdap = tetanus-diphtheria-acellular pertussis vaccine; MenACWY = meningococcal conjugate vaccine; HPV = human papillomavirus; NA = not available (estimate not reported because unweighted sample size for the denominator was <30 or 95% CI half-width/estimate > 0.6).

* Vaccination estimates for additional measures, including ≥2 doses measles-mumps-rubella vaccine, ≥3 doses hepatitis B vaccine, and ≥1 and ≥2 doses varicella vaccines are available at http://www.cdc.gov/vaccines/imz-managers/coverage/nis/teen/data/tables-2014.html.

† Adolescents (N = 20,827) in the 2014 NIS-Teen were born during the period January 1996-February 2002.

§ ≥1 dose Tdap at or after age 10 years.

¶ ≥1 dose of MenACWY or meningococcal-unknown type vaccine.

** ≥1 dose of HPV vaccine, either quadrivalent (4vHPV) or bivalent (2vHPV). Although only 4vHPV was recommended for use in males in 2014, some males might have received 2vHPV. For ≥1, ≥2, and ≥3 dose measures, separate percentages are reported among females only (N = 10,084) and among males only (N = 10,743).

†† ≥2 doses of HPV vaccine, either 4vHPV or 2vHPV.

§§ ≥3 doses of HPV vaccine, either 4vHPV or 2vHPV.

¶¶ Estimates with 95% CI half-widths >10 might not be reliable.

*** Statistically significant (p<0.05) percentage point change from 2013. The revised NIS-Teen 2013 estimates used as the basis for this comparison were calculated by retrospectively applying the revised adequate provider data definition implemented in 2014 to 2013 NIS-Teen data and, as a result, differ from those previously published. Revised NIS-Teen 2013 data included 18,948 adolescents (9,042 females and 9,906 males). Revised 2013 NIS-Teen estimates by state and selected local areas are available at http://www.cdc.gov/vaccines/imz-managers/coverage/nis/teen/apd-report.html.

††† Range excludes selected local areas and Puerto Rico.

## Footnotes

* Eligible participants were born during January 1996–February 2002. Except as noted, coverage estimates for ≥1 and ≥2 varicella vaccine doses were obtained among persons with no history of varicella disease. HPV vaccination coverage does not distinguish between bivalent (2vHPV) or quadrivalent (4vHPV) vaccines. Although the nine-valent HPV vaccine was licensed in December 2014 and routinely recommended by ACIP in February 2015 (2), the vaccine was not distributed until 2015 and therefore was not administered to adolescents in 2014. Some adolescents, both males and females, might have received more than the 3 recommended HPV vaccine doses. Influenza vaccination coverage data are not included in this report but are available online at http://www.cdc.gov/flu/fluvaxview/index.htm.

† NIS-Teen 2013 estimates provided in this report differ from those previously published (3). In 2014, NIS-Teen implemented a revised adequate provider data (APD) definition. For 2014 NIS-Teen and future surveys, any adolescent for whom one or more providers report vaccination history data or who by parental report are completely unvaccinated will be classified as having APD. Adolescents meeting either of these criteria will be included in the NIS-Teen sample and will contribute to vaccination coverage estimates. Before 2014, the APD definition was more restrictive. Adolescents had to meet one or more of the following criteria: 1) if the parent/guardian used a shot card during the household interview: have at least as many doses of measles-containing, varicella, hepatitis A, hepatitis B, and Td/Tdap vaccines by provider report as reported in the household interview, or, if a shot card was not used during the household interview: the parent/guardian indicated that the adolescent had received all vaccinations in any of the measles-containing, varicella, hepatitis A, or hepatitis B categories and the adolescent had two or more unique vaccination dates by provider report; 2) be up-to-date by provider report with ≥1 Td/Tdap, ≥3 hepatitis B, ≥2 MMR, and ≥1 varicella vaccine doses (or parental/provider report of varicella disease history); or 3) be completely unvaccinated by parental report. Questions about MMR, varicella, hepatitis A, and hepatitis B vaccines were removed from the household questionnaire in 2014. Thus, comparisons of household and provider-reported vaccination history were no longer possible for these vaccines. For this report, the revised APD definition was applied retrospectively to 2013 NIS-Teen data, resulting in 684 additional adolescents being included in the 2013 NIS-Teen sample for the United States, excluding territories, for a total of 18,948 adolescents. Additional information on implementation of the revised APD definition and an assessment of impact on vaccination coverage estimates are available at http://www.cdc.gov/vaccines/imz-managers/coverage/nis/teen/apd-report.html.

§ Local areas that received Federal Section 317 immunization funds were sampled separately: Chicago, Illinois; New York, New York; Philadelphia County, Pennsylvania; Bexar County, Texas; and Houston, Texas. One local area (El Paso County, Texas) was oversampled. One territory (Puerto Rico) was sampled separately.

¶ All identified cell phone households were eligible for interview. Sampling weights were adjusted for dual-frame (landline and cell phone), nonresponse, noncoverage, and overlapping samples of mixed telephone users. A description of NIS-Teen dual-frame survey methodology and its effect on reported vaccination estimates is available at http://www.cdc.gov/vaccines/imz-managers/coverage/nis/child/dual-frame-sampling.html.

** The Council of American Survey Research Organizations (CASRO) response rates for the landline and cell phone samples were 60.3% and 31.2%, respectively. For completed interviews in the states and local areas, 11,243 landline calls (57.1%) and 9,584 cell phone calls (52.3%) had adequate provider data. Overall, 54% of completed interviews with adequate provider data were from landlines, and 46% were from cell phones. For Puerto Rico, the landline and cell phone sample CASRO rates were 56.6% and 35.2%, respectively. The CASRO response rate is the product of three other rates: 1) the resolution rate (the proportion of telephone numbers that can be identified as either for business or residence); 2) the screening rate (the proportion of qualified households that complete the screening process); and 3) the cooperation rate (the proportion of contacted eligible households for which a completed interview is obtained).

†† Adolescents from Puerto Rico (107 females and 123 males) were excluded from the national estimates.

§§ The completion rate for 3-dose HPV vaccination series represents the percentage of adolescents who received ≥3 doses among those who had ≥1 HPV vaccine dose and ≥24 weeks between the first dose and the interview date.

¶¶ Adolescents were classified as below federal poverty level if their total family income was less than the federal poverty level specified for the applicable family size and number of children aged <18 years. All others were classified as at or above the poverty level. Poverty status was unknown for 714 adolescents. Additional information available at http://www.census.gov/hhes/www/poverty/data/threshld/index.html.

*** Seven states had ≥2-dose MMR coverage estimates among adolescents aged 13–17 years <90% and 95% CI upper bounds <90%: Arizona, Idaho, Missouri, Montana, Texas, Utah, and West Virginia. State and selected local area ≥2-dose MMR estimates are available at http://www.cdc.gov/vaccines/imz-managers/coverage/nis/teen/data/tables-2014.html.

††† Additional information available at http://www.amstat.org/meetings/jsm/2012/onlineprogram/abstractdetails.cfm?abstractid=304324.
